# Supplementary material for: A Comparative Analysis of Robot-Assisted Laparoscopic Pyeloplasty in Pediatric and Adult Patients: Does Age Matter?
Source: J Clin Med. 2022 Sep 25;11(19):5651. doi: 10.3390/jcm11195651 (PMC9570754; doi:10.3390/jcm11195651)
Supplement: Supplementary file 1 [file jcm-11-05651-s001.zip › jcm-1925781-supplementary.pdf]

Supplementary Table S1. Perioperative and postoperative outcomes according to the age groups.

	Infant group (n = 6)	Child, Adolescent group (n = 42)	Adult group (n = 69)	*P*-value
Total operative time, med (range), (mins)	134 (100–210)	176 (70–324)	148 (65–370)	0.024
Console time, med (range), (mins)	87 (60–150)	127 (78–220)	110 (86–170)	0.352
Anastomosis time, med (range), (mins)	70 (60–80)	62 (15–100)	45 (32–65)	0.144
Etiology (%)				0.052
Intrinsic-primary	6 (100)	23 (54.8)	34 (49.3)	
Intrinsic-polyp	0 (0)	6 (14.3)	5 (7.2)	
Crossing vessel	0 (0)	13 (31.0)	30 (43.5)	
Postoperative split renal function, med (range), (%)	45.7 (36.7–55.0)	41.3 (11.6–57.5)	38.9 (12.0–59.8)	0.586
Postoperative s-Creatinine, med (range), (mg/dL)	0.3 (0.2–0.3)	0.6 (0.3–1.1)	0.8 (0.5–1.6)	0.000
Pyeloplasty method (%)				0.064
Dismembered	0 (0)	3 (7.1)	0 (0)	
Nondismembered	6 (100)	39 (92.9)	69 (100)	

Surgical approach (%)				0.018
Transmesenteric	4 (66.7)	18 (42.9)	16 (23.2)	
Nontransmesenteric	2 (33.3)	24 (57.1)	53 (76.8)	
Hospital day, med (range), (day)	3.3 (2–4)	3.8 (2–12)	4.7 (3–13)	0.006
Pain score ≥ 4 requires analgesics (%)				
Postop day 0	2 (33.3)	15 (35.7)	46 (66.7)	0.003
Postop day 1	1 (16.7)	3 (7.1)	23 (33.3)	0.000
Postop day 2	1 (16.7)	0 (0)	4 (5.8)	0.001
Morphine dose, med (range), (mg/kg)				
Post op day 0	0.05 (0–0.13)	0.08 (0–0.25)	0.15 (0–0.27)	0.000
Postop day 1	0 (0)	0.09 (0–0.33)	0.24 (0–0.40)	0.000
Postop day 2	0 (0)	0.04 (0–0.30)	0.11 (0–0.46)	0.000
*Complications ≥ G3	0	3	3	
Secondary procedures needed	0	3	3	
Surgical failure (%)				0.784
Redo	0 (0)	0 (0)	1 (1.4)	
Aggravation of hydronephrosis	0 (0)	1 (2.3)	1 (1.4)	
Decrease of split renal function in 36 months	0 (0)	1 (2.3)	0 (0)	

Urolithiasis after pyeloplasty (%)				0.639
ESWL	0 (0)	2 (4.6)	1 (1.4)	
RIRS	0 (0)	1 (2.3)	1 (1.4)	

* Clavien–Dindo classification grade ≥3; ESWL, extracorporeal shock wave lithotripsy.
